# Morphohistochemical alterations of neurons of the supraoptic nucleus of the rat hypothalamus at different durations of the photoperiod and melatonin administration

**DOI:** 10.25122/jml-2021-0220

**Published:** 2021

**Authors:** Roman Yevgenovych Bulyk, Oleksiy Vasyliovych Smetanyuk, Kateryna Vasylivna Vlasova, Mariana Ivanivna Kryvchanska, Vladyslav Romanovych Yosypenko, Volodymyr Leonidovych Voloshyn, Kateryna Yuriivna Tymchuk, Tetyana Sergiivna Bulyk, Larysa Vasylivna Rynzhuk, Michael Ivanovych Sheremet, Dmytro Volodymyrovych Proniaiev

**Affiliations:** 1. Department of Medical Biology and Genetics, Bukovinian State Medical University, Chernivtsi, Ukraine; 2. Department of Obstetrics and Gynecology, Bukovinian State Medical University, Chernivtsi, Ukraine; 3. Surgery Department No.1, Bukovinian State Medical University, Chernivtsi, Ukraine; 4. Department of Anatomy, Clinical Anatomy and Operative Surgery, Bukovinian State Medical University, Chernivtsi, Ukraine

**Keywords:** hypothalamus, supraoptic nucleus, photoperiod, melatonin

## Abstract

We studied the morphologic and histochemical organization of neurons of the hypothalamic supraoptic nucleus in rats exposed to different durations of photoperiod and injection of melatonin. Morphometric and histochemical analyses of neurons were performed after staining brain histological sections for RNA. Prolonged illumination leads to more pronounced changes in the parameters of hypothalamic structures at 2 a.m. than at 2 p.m., particularly decreasing the concentration of RNA in the cell nuclei. The use of exogenous melatonin does not normalize the revealed changes in the parameters of the studied structures of the neurons of the supraoptic nucleus of the hypothalamus caused by the prolonged stay of rats under conditions of constant illumination.

## Introduction

The brain is the central organ of systemic adaptation to various stressors because it determines the strategy of neurophysiological, neuroendocrine, and behavioral reactions, which can be protective or destructive for the body under the action of acute or chronic stress [1–4]. Brain reactions are associated with the stimulation of hypothalamic-pituitary-adrenocortical and autonomic nervous systems, the regulatory influence of these systems on metabolism, and the activity of pro-and anti-inflammatory components of the immune system [5–7]. Supraoptic nuclei (SON) are one of the main components of the hypothalamic neuroendocrine system, which provide a synthesis of vasopressin and oxytocin and transport to the neurohypophysis, then to the bloodstream. Vasopressin is involved in the regulation of water-salt balance. Furthermore, it also provides neuroendocrine pathways regarding the regulation of circadian changes, ensuring the formation of biological rhythms in almost all members of the animal and human world. [8–11]. The alternation of the circadian cycle of day and night is the most important regulator of various physiological rhythms in all living organisms [12–14]. The invention of electricity and artificial light more than a century ago radically changed the light regime and the duration of exposure to light. The impact of light at night, often called light pollution, has increased and has become an essential part of modern life, accompanied by many serious behavioral and health disorders. Both suprachiasmatic nucleus (SCN) of the anterior hypothalamus and pineal gland are a part of the “biological clock” and play a pivotal role in the mechanisms of “counting the internal time” [15–19]. At the same time, the hypothalamic SCN plays the part of a central oscillator regulating the adjustment of metabolic and energy rhythms to the rhythms of illumination as an exogenous energy source [20–23]. The coding of light mode information is performed by the main hormone of the pineal gland, melatonin [24, 25]. Melatonin is the main regulator of seasonal and circadian rhythms, impacting the functional activity of hypothalamic-pituitary-adrenal and reproductive systems. Secretion of melatonin is associated with circadian rhythms; the highest values are recorded at 2 a.m., while its concentration decreases to minimum values during the day. Changing the lighting mode (light stimulation or light deprivation) is a determining stress factor, which leads to the evolution of desynchronosis [26, 27]. Given the heavy role of large-cell neurosecretory supraoptic nuclei of the anterior hypothalamus in the implementation of the body adaptive capacity, it is appropriate to determine the character of their rejoinder among experimental animals exposed to different photoperiods and the possible impact of melatonin on morpho-histochemical changing of the supraoptic nucleus [28].

## Material and Methods

The goal of the research was to investigate the peculiarities of morpho-histochemical characteristics of neurons of the SON of the hypothalamus of mature rats under different light modes and injection of melatonin. The experiments were performed on 36 mature male outbred white rats weighing 150–180 g. The animals were kept in standard retainer conditions at constant temperature and humidity and had free access to water and food. Experimental rats were divided into three groups, each of which, in turn, consisted of two subgroups (six animals each).

The first group (control) represented animals kept under standard light mode for seven days (light/dark for 12 hours, LD, lighting from 8 a.m. to 8 p.m. with fluorescent lamps, illumination level in the animal cages of 500 lx). The second group included rats under constant lighting or light stimulation conditions for seven days (LL, simulation of pineal gland hypofunction). Animals in the third group were kept under the same experimental conditions as the rats in the second group. In addition, these rats were intraperitoneally injected with melatonin (Sigma, USA, 99.5% purity) daily at 7 p.m. with a dose of 0.5 mg/kg in 1.0 ml of solvent (0.9% ethanol solution on normal saline) (LL + melatonin). After the end of the seven-day experiment, the animals were removed from the experiment by one-step decapitation. In order to reveal the morphofunctional differences of the studied structures and take into account the cyclicity of melatonin production, the biomaterial was collected at 12-hour intervals (at 2 p.m. and 2 a.m.). The brain from the animals in the control and experiment groups was immediately extracted, fixed in Carnoy’s fluid, and embedded in paraffin after standard histological treatment. To study morphometric parameters of neurons in the SON of the hypothalamus, histological sections 7 μm thick were deparaffinized in xylene, rehydrated in descending concentrations of ethanol (100%, 96%, 70%), washed three times by distilled water, and stained by Einarson method in halocyanine-chrome alum solution for 48h, which allows the discovery of nucleic acids (mainly RNA) in neurons. Sections were then washed three times in distilled water, dehydrated in ascending ethanol concentrations (70%, 96%, 100%), xylene, and placed in Canadian balsam. Morphometric analysis of neurons was carried out by a computer system for digital image evaluation VIDAS-386 (Kontron Elektronik, Germany) in the visible spectrum. The image obtained with an Axioskop microscope using a COHU-4922 video camera (COHU Inc., USA) was transferred into the VIDAS-386 computer system for digital image analysis (Kontron Elektronik, Germany). Image analysis was implemented using the VIDAS-2.5 application software package (Kontron Elektronik, Germany) in semi-automated mode. At least 100 cells in each series of studies were analyzed, for which we determined the morphometric parameters of neurons – the area and equivalent diameter of cells, their nuclei, nucleolus, and cytoplasm, and the densitometric characteristic – the optical density (in conventional units of optical density, absorbance units, AU) of cell nuclei, nucleolus, and cytoplasm, which were conditioned by the level of RNA accumulation. The experimental data were processed using the PC application package program VIDAS-2.5 (Kontron Elektronik, Germany) and Excel (Microsoft Corp., USA). The arithmetic means of the sample (x), its alternation, and mean error (Sx) were calculated for all indices. In order to determine the reliability of differences in the results of research in the control and experimental groups of animals, Student’s coefficient (t) was determined.

## Results

A study of the morphometric parameters of supraoptic hypothalamic neurons in control animals (LD group) at 2 p.m. revealed that the neuron body area was 273.89±4.298 μm^2^, nucleus 74.47±1.262 μm^2^, nucleolus 31.05±4.448 μm^2^ and cytoplasm 199.42±4.172 μm^2^ (Table 1). The nucleus-cytoplasmic ratio (NCR) was within 2.62±0.022 units. The specific volume of the neuron nucleus was 27.64±0.561%, and the specific volume of the cytoplasm was 72.36±0.562%.

**Table 1.** Morphometric indices of the rat supraoptic nucleus of hypothalamus neuron in rats at different diurnal periods (x̄±S_x̄_).

**Animal group (subgroup)**		**Neuron area, μm^2^**	**Neuron nucleus area, μm^2^**	**Neuron nucleolus area, μm^2^**	**Cytoplasm area, μm^2^**
**1**	**LD, 2 p.m.**	273.89±4.298	74.47±1.262	31.05±4.448	199.42±4.172
	**LD, 2 a.m.**	305.67±7.939 p<0.01	87.70±4.016 p<0.05	36.68±8.804	217.98±5.930 p<0.05
**2**	**LL, 2 p.m.**	294.89±6.369	94.08±8.546 p_1_<0.05	47.61±6.184 p_1_<0.05	200.82±9.071
	**LL, 2 a.m.**	306.50±11.338	103.39±5.051 p^1^<0.05	68.13±8.970 p^1^<0.05	203.11±7.101
**3**	**LL + melatonin, 2 p.m.**	300.79±11.539 p<0.01	96.38±6.203 p<0.05	46.09±8.177 p<0.01	204.41±7.824
	**LL + melatonin, 2 a.m.**	308.25±9.959	106.89±5.514 p_1_<0.05	71.01±7.013 p<0.05 p^1^<0.01	201.36±6.881

p – probable changes relative to the parameters of the animals of the previous time interval in the group; p_1_ – probable changes relative to the parameters of the animals of the LD subgroup of the same time interval.

Determination of morphometric parameters of the hypothalamic SON neurons revealed circadian dynamics of the indices. We reveal a reliable 11.6% increase in the neurons body area of the hypothalamic SON at 2 a.m., caused by an enlargement in the nucleus area of the cell of 17.8% in comparison with the daily period (2 p.m.) in the LD group. Moreover, the enlarged nucleus area of the neurons was caused by the rise of its nucleolus area, which was 36.68±8.804 μm^2^ (Table 1). At the same time, the NCR in vasopressin-synthesizing structures was 2.91±0.019% and significantly greater (by 11.1%) during nighttime observation than during the daytime interval. Equally, the specific volume of neuron cytoplasm increased by 2.8%, while the nucleus, on the contrary, decreased by 7.3%. These changes were associated with expanding RNA concentration in nuclei of the neurons at 2 a.m. compared to 2 p.m. on a 13.2%. Simultaneously, we did not observe any essential changes in RNA concentration in the cytoplasm of the SON of the hypothalamus neurons (Table 2). The data obtained indicate a promotion in the functionary and synthetic activity of neurons of the hypothalamic SON in LD rats at night.

**Table 2.** RNA concentration in the nucleus, nucleolus, and cytoplasm of rat hypothalamic supraoptic neurons (x̄±S_x̄_).

Animal group (subgroup)		Nucleus RNA concentration, AU	Nucleolus RNA concentration, AU	Cytoplasm RNA concentration, AU
**1**	**LD, 2 p.m.**	0.142±0.0024	0.333±0.0028	0.071±0.0022
	**LD, 2 a.m.**	0.187±0.0077 p<0.001	0.304±0.0121 p<0.05	0.070±0.0037
**2**	**LL, 2 p.m.**	0.144±0.0073	0.329±0.0339	0.087±0.0045 p_1_<0.01
	**LL, 2 a.m.**	0.152±0.0058 p_1_<0.01	0.364±0.0234 p_1_<0.05	0.091±0.0043 p_1_<0.01
**3**	**LL + melatonin, 2 p.m.**	0.132±0.0045	0.343±0.0327	0.088±0.0043 p_1_<0.01
	**LL + melatonin, 2 a.m.**	0.131±0.0034 p_1_<0.01 p_1_<0.05	0.351±0.0151 p_1_<0.01	0.096±0.0032 p_1_<0.01

p – probable changes relative to the parameters of the animals of the previous time interval in the group; p_1_ – probable changes relative to the parameters of the animals of the LD subgroup of the same time interval; p_2_ – probable changes relative to the parameters of the animals of the LL subgroup of the same time interval.

Photoperiod (duration of daylight hours) has one of the greatest impacts on the physiological processes of organs and systems among the external environmental factors. There are no significant differences in the neuronal area of the SON between animals that were under constant light (LL group) at 2 p.m. and of the LD group (Table 1). However, we revealed an enhancement in the size of its nucleus by 26.3±2.1%. The alters in the nucleus size were evoked by an enhancement in the neuron nucleolus area, which was 47.61±6.184 μm^2^ and 53.3±3.7% larger than in the LD group. Attention was also drawn to the significant decrease in the LD group of the NCR by 4.2±0.3%, which was 2.51±0.023 units. This was evoked by an alteration in the parameter concerning growth in the specific volume of the nucleus by 28.47±0.953% and a diminish in the specific volume of the cytoplasm by 71.53±0.979%. In the meantime, the specific volume of the neuron of the nucleolus was credibly raised about twice and was 8.06±1.623% of the volume of the studied neuron.

Extended light exposure in the LL group resulted in a substantive enhancement in RNA concentration in the cytoplasm of neurons at 2 p.m., which was 0.087±0.0045 AU and was 22.54±3.7% higher than the indicator in the LD group during the same time. However, RNA amounts in the neuron nucleus and nucleolus keep near the pointer in the LD group (Table 2).

In what concerns rats in the LL group, the NCR of the SON neuron at 2 a.m. was noticeably lower than that of the LD group (by 17.18±1.14%) through an enlarged specific volume of the neuron nucleus and nucleolus, which were 29.31±0.829% and 11±1.523% in accordance, and a potential reduction in the specific volume of the cell cytoplasm, which reached 70.69±0.834%. 

At 2 a.m., we observed more expressive replacement in the morphofunctional state of the hypothalamic SON than at 2 p.m. in animals kept under conditions of round-the-clock lighting. Therefore, the neuronal nucleolus area was 68.13±8.970 μm^2^ and was appreciably larger than the LD group. Furthermore, the cytoplasmic area of the neuron was 203.11±7.101 μm^2^ and was near that of the animals in the LD group. It should be noted that animals staying in round-the-clock light conditions did not impact the circadian rhythm of the morphofunctional activity of the SON of the hypothalamus. Their higher activity, as in the LD group, was registered on nighttime observation, which was evidenced by the analysis of the obtained data of the morphometric alteration of the neurons studied. During the night phase of the research, we point out a reliable enlargement in the concentration of RNA in the nucleolus and cytoplasm of the neurons SON at 2 a.m. compared to data from the same time from animals in the LD group. This regularity was associated with a considerable decrease in RNA concentration in the neuron nucleus, which was 0.152±0.0058 AU. In this circadian interval, nucleic acid concentration did not diverge considerably compared to the animals in the LD group (Table 2). At 2 a.m., animals in the LL group showed a considerable decrease in NCR in the study neurons by 17.18±1.97% compared to the LD group in the same period and compared to the LL group of the previous time of research.

The results obtained suggest that the photoperiod duration considerably impacts the neurons of the SON of the hypothalamus in rats. Round-the-clock lighting does not cause inversion of the circadian rhythm of morphofunctional activity of the studied structures; the highest values, as in the animals in the LD group, fall in the nighttime. Keeping animals under round-the-clock lighting conditions for a long time leads to a considerable enhancement in the area of the nucleus and nucleolus of neurons compared to animals in the LD group, both during the day and the nighttime. Simultaneously, we noticed a reduction in NCR, an enhancement in RNA concentration in the nucleolus and cytoplasm of neurons of SON of the hypothalamus of rats during nighttime.

Exogenous melatonin at a dose of 0.5 mg/kg in 1.0 ml of solvent was used to correct the disorders caused by extended exposure of rats to light stimulation. When rats got hormone on the background of stress caused by round-the-clock lighting (LL + melatonin group) during the daytime, the neuronal area was 300.79±11.539 μm^2^, the nucleus 96.38±6.203 μm^2^, and the nucleolus 46.09±8.177 μm^2^. The listed parameters were somewhat larger than those of the LL group (Table 1). NCR was within the range of 2.36±0.013 AU and was considerably lower than the animals in the LD and LL groups. The nucleus-specific volume index was 29.76±0.801%, and the cytoplasm was 70.24±0.803%, with no significant difference in the comparison groups. As for RNA concentration in the structures of the hypothalamic SON cells, during the daytime interval of the study, it was 0.132±0.0045 AU in the nucleus, 0.343±0.0327 AU in the nucleolus, and 0.088±0.0043 AU in the cytoplasm. There are no reliable differences between these values of the same time of research in animals in the LL group (Table 2).

As in animals in the LL + melatonin group at 2 p.m., the enlargement in the neuron area at night was due to the enlargement of the nucleus and nucleolus area of the neurons (Table 1). Determination at 2 a.m. of NCR, specific volumes of the nucleus, nucleolus, and cytoplasm of neurons of rat hypothalamus SON showed no reliable difference in the indices from animals of the previous time interval. It must be specified that the concentration of RNA in the nucleus of the SON neurons of the hypothalamus was 0.131±0.0034 AU and was significantly lower compared to the animals in the LD and LL groups. Under these experimental conditions, the concentration of ribonucleic acid in the nucleolus and cytoplasm of the neurons was considerably higher than in the LD group, and no considerable differences were obtained with the other comparison groups (Table 2). It should be noted that, in general, the use of melatonin did not compensate for the changes in the morphometric parameters of the studied structures of the neurons of the SON of the hypothalamic caused by prolonged exposure of rats to round-the-clock light conditions.

## Discussion

It is known that vasopressin is one of the main neurohormones of the SON [1]. Moreover, it plays an important role in the activation of the pituitary-adrenocortical system in reply to stress [6]. The findings indicate that the vasopressinergic system of the hypothalamus, represented by large-cell neurocytes, is involved in the mechanisms of the neuroendocrine response of the organism to a prolonged change in the light regime. Simultaneously, the character of the disclosed changes in the morphofunctional state of the SON in response to changes of the photoperiod duration does not suggest a considerable increase in the synthesis of this neurohormone against the background of a considerable decrease in RNA concentration in the neuronal nuclei when observed at 2 a.m., as opposed to 2 p.m. The possible reasons for such a reaction of large-cell neurons of SON may be, in our opinion, the preferential involvement of SON vasopressin in the regulation of arterial pressure and water-salt metabolism. A similar pattern was demonstrated for supraoptic nucleus neurons of Wistar white rats under the influence of intermittent hypoxia [29]. Constant illumination and light deprivation are rather significant stressors [30]. Comparison of histochemical and morphometric characteristics of supraoptic hypothalamic neurons under normal photoperiodism and artificial modifications of the latter indicates that cerebral structures involved in the organization of circadian rhythm and realization of stress reactions closely interact with each other and with an important neuroendocrine mediator, brain epiphysis. Both direct and inverse essential functional connections probably exist between these structures. Obviously, this situation is associated with a rather complicated pattern of sometimes unexpected changes that occur in these CNS structures during experimental changes in the light regime and melatonin administration.

## Conclusions

The duration of the photoperiod considerably impacts the activity of neurons of the supraoptic nuclei of the hypothalamus of rats. Keeping animals under round-the-clock light conditions leads to more distinct changes in histochemical and morphometric parameters of hypothalamic structures at 2 a.m. compared to 2 p.m., diminishing RNA concentration in cell nuclei. The injection of exogenous melatonin does not make up for the changes in the parameters of the studied structures of the hypothalamic supraoptic neurons caused by prolonged exposure of rats to constant light.

## Acknowledgments

### Conflict of interest

The authors declare that there is no conflict of interest.

### Ethical approval

Ethical approval for this study was obtained from the Ethics Committee of the Bukovinian State Medical University (approval ID: 1/20.09.2018).

### Personal thanks

The authors would like to acknowledge the administration of Bukovinian State Medical University.

### Authorship

RYB and TSB contributed to conceptualizing, KVV and DVP contributed to the methodology, OVS and KYT contributed to writing the original draft, VLV and MIS contributed to editing the manuscript, VRY contributed to data collection, MYK and LVR contributed to data analysis.
